# Plasma Treatment of Polypropylene-Based Wood–Plastic Composites (WPC): Influences of Working Gas

**DOI:** 10.3390/polym12091933

**Published:** 2020-08-27

**Authors:** Philipp Sauerbier, Robert Köhler, Gerrit Renner, Holger Militz

**Affiliations:** 1 Wood Biology and Wood Products, Faculty of Forest Sciences, University of Goettingen, Büsgenweg 4, 37077 Göttingen, Germany; holz@uni-goettingen.de; 2 Laboratory of Laser and Plasma Technologies, University of Applied Sciences and Arts, Von-Ossietzky-Str. 99, 37085 Göttingen, Germany; robert.koehler@hawk.de; 3 Instrumental Analytical Chemistry, Faculty of Chemistry, University of Duisburg-Essen, Universitätsstr. 5, 5141 Essen, Germany; gerrit.renner@uni-due.de

**Keywords:** wood–polymer composites, plasma treatment, coatings

## Abstract

In this study, a polypropylene (PP)-based wood–plastic composite with maleic anhydride-grafted polypropylene (MAPP) as a coupling agent and a wood content of 60% was extruded and specimens were injection molded. The samples were plasma treated utilizing a dielectric barrier discharge (DBD) setup with three different working gases: Ar/O_2_ (90%/10%), Ar/N_2_ (90%/10%), and synthetic air. This process aims to improve the coating and gluing properties of the otherwise challenging apolar surface of PP based wood–plastic composites (WPC). Chemical analysis with X-ray photoelectron spectroscopy (XPS) and Fourier-transform infrared spectroscopy (FTIR) showed the formation of oxygen-based functional groups on the surface, independently from the working gas used for the treatment. Laser scanning microscopy (LSM) examined the surface roughness and revealed that the two argon-containing working gases roughened the surface more than synthetic air. However, the contact angle for water was reduced significantly after treatment, revealing measurement artifacts for water and diiodomethane due to the severe changes in surface morphology. The adhesion of acrylic dispersion coating was significantly increased, resulting in a pull-off strength of approximately 4 N/mm^2^, and cross-cut tests assigned the best adhesion class (0), on a scale from 0 to 5, after plasma treatment with any working gas.

## 1. Introduction

Wood–plastic composites (WPCs) have been more common in commercial products throughout the last decades. They offer the possibility of decreasing the share of often fossil-based polymers while substituting them with high amounts of cheap and sustainable wood flour. They also provide good physical properties [1,2] and environmental benefits [3,4], especially when upcycled and recycled materials are being used [5,6].

Alkene-based WPCs, namely polypropylene (PP) and polyethylene (PE), are the most widespread on the market. Deckings for outdoor flooring and the automotive industry are the main use cases, and smaller shares are used for consumer goods such as toys [7,8]. Due to the matrix polymer’s apolar properties, coating or printing processes bear challenges. The same is true for gluing parts together, either to repair a broken part or more recently to assemble (large) 3D-printed ones. In most cases, these challenges are addressed with a pre-treatment, either by additives [9] or a direct treatment of the surface [10].

Low-temperature atmospheric-pressure plasma has become one of the industry standards for all kinds of surface activations and alterations. This is especially true for the highly alkene-focused polymer industry. A plasma treatment addresses the apolarity, causing an increase in hydrophilicity and therefore improving wettability and paint/glue adhesion [11,12].

More recently, plasma treatments of wooden and wood-based products have been investigated, e.g., veneers for laminated veneer lumber, particleboards, and solid wood [13,14,15,16]. The results of Hünnekens et al. show a positive effect of a low-temperature atmospheric-pressure plasma for extruded and injection-molded specimens alike [17]. The comparison of different surface activation methods for WPC revealed a superior surface activation even when compared to other methods, e.g., adhesion promoter, etching, or flaming [18,19,20].

The presented study builds up on these findings. While previous studies used ambient air for the plasma treatment, three different well-defined working gases have been used in this study to investigate the effects of the plasma treatment on self-produced PP-based WPC with a wood content of 60%. To gain a better understanding of the plasma treatment’s modes of action, X-ray photoelectron spectroscopy (XPS) and Fourier-transform infrared spectroscopy (FTIR) are used to investigate expected changes, e.g., the formation of functional groups, in the surface chemistry after the plasma treatment. Laser scanning microscopy (LSM) is utilized to describe the changes to the surface morphology, as a surface roughening is reported in the cited literature. Following a more practicable approach, secondary changes induced by the plasma treatment such as the wettability are investigated with the contact angle measurements of water and diiodomethane as well as the paint adhesion with the determination of the pull-off strength and performing a cross-cut test.

With the combination of practical and fundamental research methods, the authors aim to contribute to a better understanding of the effects caused by the plasma treatment on WPCs as well as improvement of their practical application.

## 2. Materials and Methods

### 2.1. Specimen Preparation

The WPC was manufactured using a co-rotating twin-screw extruder MICRO27GL/GG40D (Leistritz, Nürnberg, Germany) equipped with gravimetric feeders (Brabender Technology GmbH, Duisburg, Germany) and a pelletizer. The average melting temperature at the die was kept at around 140 °C. Then, the granules were used to produce test specimens by injection molding with an ALROUNDER 420 C (Arburg, Loßburg, Germany). The temperature was kept at approximately 110–115 °C with a total cycle time of 40 s.

Isotactic polypropylene (PP) (Sabic 575P, Riyadh, Saudi Arabia) was used as polymer matrix with 37% by weight of the final compound. Softwood flour Arbocel^®^ C100 with a particle size of 30–200 µm (J. Rettenmaier & Söhne, Rosenberg, Germany) was added with respectively 60% by weight as well as maleic anhydride-grafted polypropylene (MAPP) Licocene 7452 TP (Clariant, Muttenz, Switzerland) as coupling agent with 3 wt %. (The formulation will be abbreviated PP60 for the remaining publication.)

For the tests, the specimens were cut into pieces of (100 × 50 × 4) mm^3^ for the pull-off test and (50 × 50 × 4) mm^3^ for all other analysis methods. The samples were stored in normal climate (20 °C, 65% relative humidity) to ensure a standardized conditioning. Additionally, prior to the treatment, all samples, including the untreated references, were cleaned with 2-propanol.

### 2.2. Plasma Treatment

The plasma treatment of the specimens was performed using a dielectric barrier discharge (DBD) setup. The specimens were placed on glass to insulate them from the ground electrode underneath. The upper electrode was positioned 2 mm above the specimen, and the resulting gap was flooded with the working gas with a flow of 150 L/min. Three different working gases were tested: oxygen/argon (10%/90%), nitrogen/argon (10%/90%), and synthetic air (80% nitrogen/20% oxygen).

The samples were plasma treated with an applied peak-to-peak voltage (pulsed sine bipolar high voltage, pulsed duration of approximately 1.9 µs) of approximately 24 kV and a repetition frequency of 17 kHz for a net treatment time of 30 s, with 1 s of treatment followed by 1 s of non-treatment (total of 60 s). This was done to ensure that that the specimen’s surface stays below 60 °C as measured in a previous study using the same plasma device [21].

### 2.3. Analysis

#### 2.3.1. Fourier-Transform Infrared Spectroscopy (FTIR)

FTIR measurements of five samples each variant were performed on a PerkinElmer Frontier (PerkinElmer LAS (Deutschland) GmbH, Rodgau Jügesheim, Germany). The measurement was performed with diamond ATR (Specac Golden Gate GS 10515, Specac Ltd., Orpington, UK) in a range of 400–4000 cm^−1^, averaged over 64 scans with a resolution of 4 cm^−1^.

#### 2.3.2. X-ray Photoelectron Spectroscopy (XPS)

The measurements were performed on a PHI 5000 Versa Probe II (ULVAC-PHI, Chigasaki, Japan) using monochromatic Al-K_α_ radiation with 1486.6 eV of photon energy. The minimum detector resolution measured at the Ag3d 5/2 peak is 0.45 eV. Detailed spectra of carbon 1s (C1s), oxygen 1s (O1s), and nitrogen 1s (N1s) with a pass energy of 46.95 eV, a step size of 0.1 eV, and a spot size of 200 µm were collected for three samples of each variant. In order to avoid charging effects, the measurements were carried out with the neutralization of sample charging. The structures were fitted applying Voigt profiles after conducting a Shirley baseline subtraction.

#### 2.3.3. Contact Angle

The contact angle of an apolar (diiodomethane) and a polar liquid (water) on the sample’s surface was measured with a Krüss G10 (Krüss GmbH, Hamburg, Germany). Diiodomethane droplets with a volume of 7 µL and water droplets of 10 µL were dosed, and the resulting angles were analyzed using the corresponding software DSA 1 1.90 (Krüss GmbH, Hamburg, Germany) with the Tangent 1 fit. For this fit, an elliptical drop shape is assumed. It fits a conic section to the drop shape, and the contact angle is determined as the angle between the baseline and the tangent at the conic section curve at the three-phase contact point [22]. The obtained data correspond with the surface free energy, separated in a polar and a disperse part, that can be calculated following the approach of Owens, Wendt, Rabel, and Kaelbe (OWRK) [23,24,25].

For each variant, five samples were analyzed with 12 droplets of each liquid per sample.

#### 2.3.4. Laser Scanning Microscopy (LSM)

The KEYENCE VK-X100 (KEYENCE Deutschland GmbH, Neu-Isenburg, Germany) with an 100× objective was used for the roughness measurements of the samples.

Five samples each variant were analyzed by measuring 12 (3 × 4) single adjacent images (135 × 101 µm^2^) and stitching them together to the size of approximately 400 × 400 µm^2^. On this area, 10 squares in the size of 20 × 20 µm^2^ were randomly placed, and with the resulting data, the developed interfacial area ratio (Sdr) was calculated according to DIN EN ISO 25178 (2016) [26] without adding any additional filter. To ensure conclusive results and reliable surface data, the specimens were measured at the same position, before and after treatment.

#### 2.3.5. Paint Adhesion/Pull-Off Strength/Cross-Cut Test

To analyze the paint adhesion of an acrylic dispersion, the samples were roll painted with Alpina Weißlack für Innen (Alpina Farben GmbH, Ober Ramstadt, Germany). In total, three layers of coating were applied, each after 24 h of drying, with the first layer being put on within one hour after plasma treatment.

The pull-off strength was determined using the dolly test based on ASTM D4541-02 (2009) and DIN EN ISO 4624 (2016) [27,28]. In total, 15 dollies (three each (100 × 50 × 4) mm^3^ specimen) with a diameter of 20 mm for each variant were glued on the coating’s surface with Araldite 2011 (Huntsman Advanced Materials LLC, Salt Lake City, UT, USA). The extraction was performed with a hydraulic hand-held measuring device PosiTest AT-P (DeFelsko Corporation, Ogdensburg, NY, USA).

The cross-cut test followed DIN EN ISO 2409 (2013) [29], and for each variant, three samples were cross-cut at three different locations. Since the coating’s thickness was determined with a digital microscope (KEYENCE VHX-5000 KEYENCE Deutschland GmbH, Neu-Isenburg, Germany) to be (47 ± 5) µm, a 1 mm grid was used for the cuts, as required by the standard.

#### 2.3.6. Statistical Analysis

A Kolmogorov–Smirnov normality test was used to ensure a normal distribution (α = 0.05). The data were further analyzed with a two-sample unequal variance (heteroscedastic) t-Test (α = 0.05) and/or a Tukey HSD (honestly significant difference) test (α = 0.05) to test the results for statistical differences between the variants.

## 3. Results

The data from the various analysis methods can be found in the following subsections.

### 3.1. Fourier-Transform Infrared Spectroscopy (FTIR)

**Figure 1:**
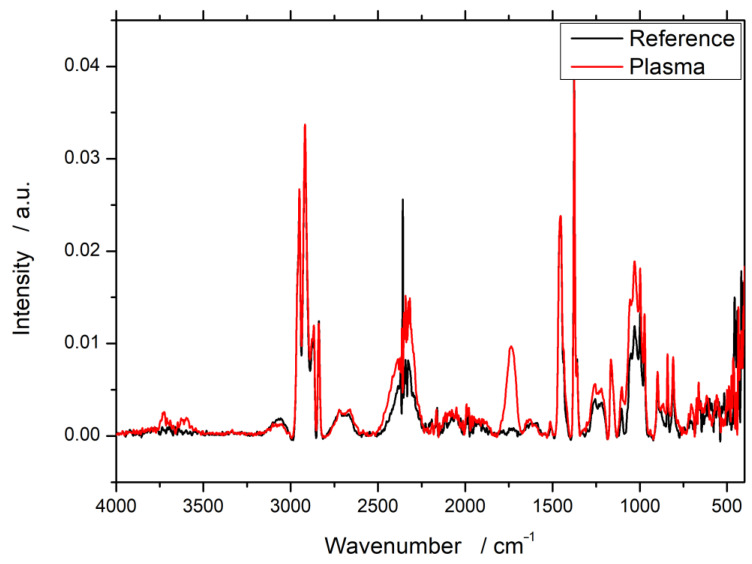
Fourier-transform infrared spectroscopy (FTIR) spectra of wood–plastic composites (WPC) with 60% wood content in polypropylene (PP) before and after plasma treatment with 90% argon and 10% oxygen as working gas.

**Figure A1:**
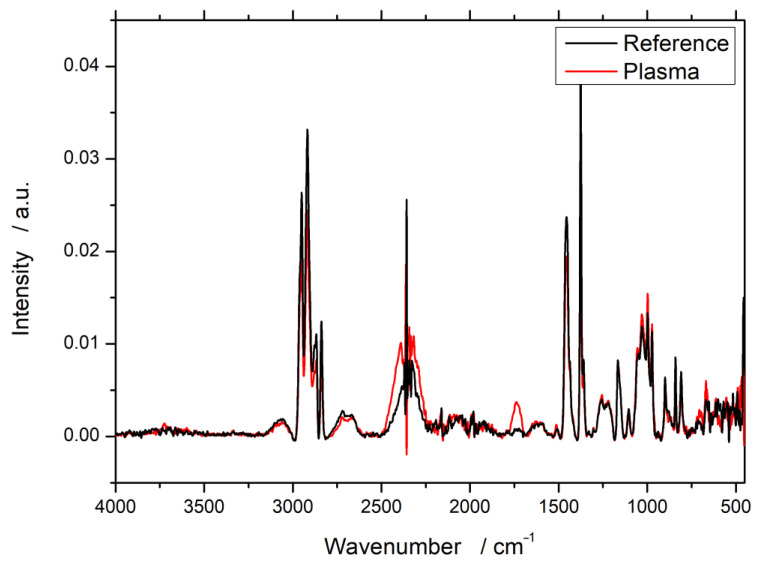
FTIR spectra of WPC with 60% wood content in PP before and after plasma treatment with 90% argon and 10% nitrogen as working gas.

**Figure A2:**
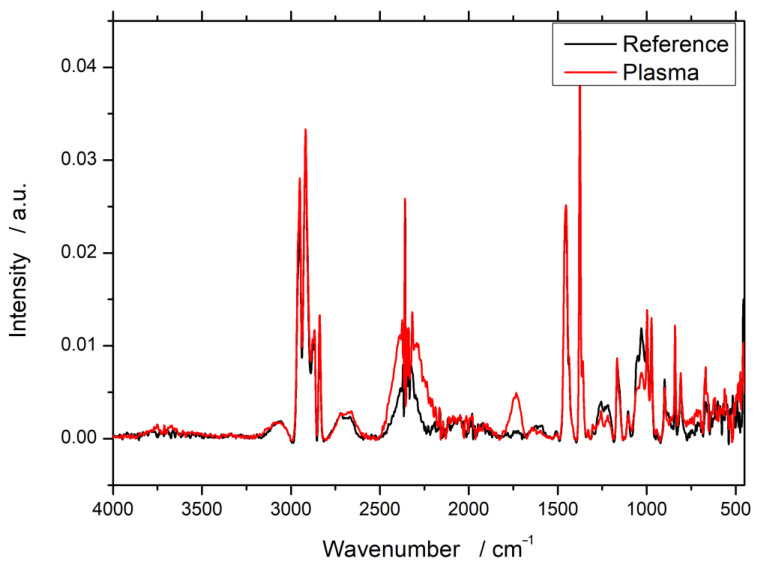
FTIR spectra of WPC with 60% wood content in PP before and after plasma treatment with synthetic air as working gas.

The FTIR spectroscopy data show comparable results with no particular influence of the working gas used for the plasma treatment. As an example, the results for PP60 after treatment with Ar/O_2_ are shown in Figure 1. The spectra for Ar/N_2_ and synthetic air can be found in the Appendix A (Figure A1 and Figure A2).

Due to the only minor quantifiable nature of FTIR measurements, only new peaks are of a clear meaning. In all cases, a minor but broad peak increase in the range of 3600 wavenumbers, which can be assigned to hydroxyl groups, can be observed. However, the clearest difference is at about 1750 cm^−1^. These bands can be assigned to double bound oxygen, typically from carbonyl groups. This newly formed peak at 1750 cm^−1^ is visible for all three working gases, even the one without oxygen (Ar/N_2_).

### 3.2. X-ray Photoelectron Spectroscopy (XPS)

**Figure 2:**
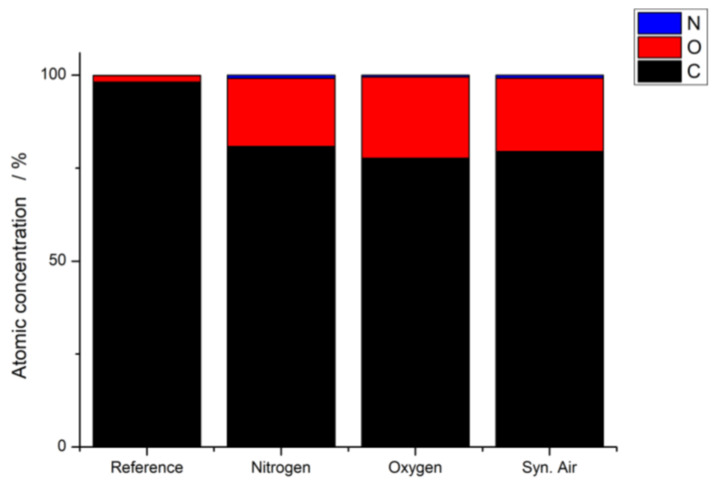
Elemental composition (Nitrogen, Oxygen and Carbon) of WPC with 60% wood content in PP before and after plasma treatment with 90% argon and 10% nitrogen as working gas.

The XPS measurements allow analyzing the elemental composition of a specimen’s surface. Similar to the FTIR data, the XPS obtained data showed the same results independent of the working gas. As an example, Figure 2 and Table 1 show the elemental composition of a substrate after plasma treatment with Ar/N_2_ as the working gas.

It is evident that newly formed oxygen-containing functional groups have been formed due to the plasma treatment for all working gases. While the atomic concentration of carbon is decreased, the oxygen content rose almost exactly by the same amount: only minor and negligible changes for nitrogen could be observed.

**Figure 3:**
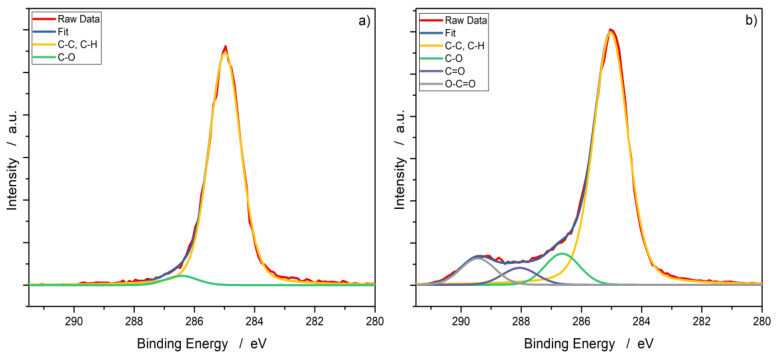
XPS data of the carbon peak with fitted Voigt profiles of PP60 (**a**) before and (**b**) after plasma treatment with Ar/N_2_ as the working gas.

Figure 3 shows the C1s peak of a PP60 specimen before and after the plasma treatment (exemplified again, plasma treated with Ar/N_2_). The spectra were shifted to the main peak of C1s at 285.0 eV [30]. For the reference, two structures are recognizable: the main peak can be assigned to C–C and C–H. The second structure around 286.6 eV can be attributed to C–O, which is due to the presence of low-oxidation carbon on the surface [31]. In the plasma-treated samples, four structures are visible. The first two correspond to the same peaks as described for the reference. The peaks around 287.9 eV and 289.3 eV can be assigned to double bound oxygen—to be more precise, C=O and O–C=O [32]. A more in-depth listing of the fits and percent distribution of the fitted carbon peaks can be found in Table A1 in the Appendix A.

### 3.3. Contact Angle

**Figure 4:**
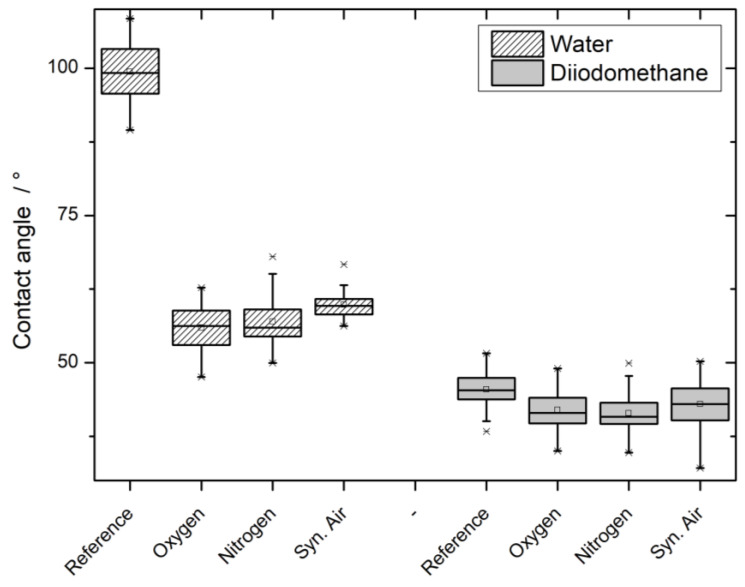
Contact angles of water and diiodomethane on PP60 before and after plasma treatment.

**Figure A3:**
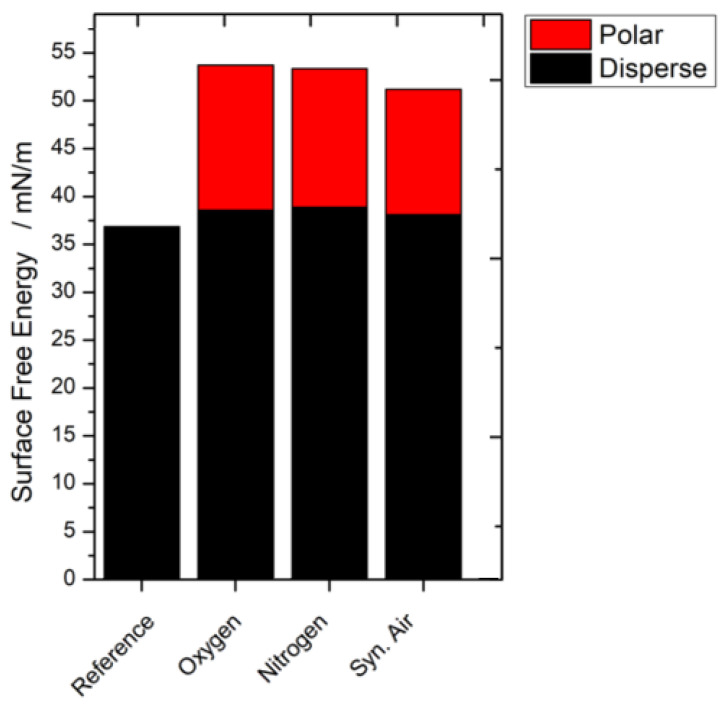
Calculated surface free energy according to the Owens, Wendt, Rabel, and Kaelbe (OWRK) method based on contact angle results of WPC with 60% wood content in PP before and after plasma treatment. For all three working gases, the surface free energy was significantly increased due to a newly formed polar part, due to new functional groups on the surface.

The surface free energy according to OWRK was calculated, and the results can be found in Figure A3 in the Appendix A. However, the raw data of contact angles of water and diiodomethane will be used for the discussion, as measurement artifacts are easier to detect and discuss in this form. The visualized data can be found in Figure 4.

Evidently, the initially high water contact angle of PP-based WPC of around 100° is lowered to an angle in the range of 50–60°. The observed effect significantly decreases the contact angle more for the nitrogen and oxygen working gases compared to synthetic air. The reduction of contact angle can also be observed for diiodomethane droplets, but on a smaller scale. Oxygen and nitrogen-based working gases again reduce the contact angle more, when compared to synthetic air. However, this difference is not statistically significant.

### 3.4. Laser Scanning Microscopy (LSM)

**Figure 5:**
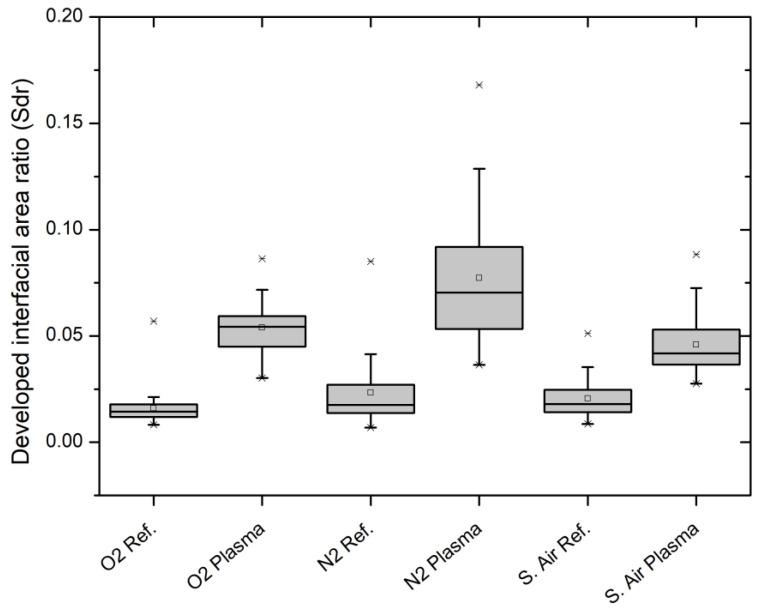
Developed interfacial area ratio (Sdr) of references and plasma treated PP60 specimens.

The developed interfacial area ratio (Sdr) was calculated according to DIN EN ISO 25178. The visualization can be found in Figure 5.

The surface area of all WPC specimens was increased for all three working gases due to the plasma treatment. Since all samples were analyzed before and after the treatment, the calculation of percentual area growth of the Sdr is possible. Given the higher initial mean surface roughness for the samples later treated with synthetic air plasma, the obtained growth is significantly lower in comparison to the other two treatments (Table 2).

### 3.5. Paint Adhesion/Pull-Off Strength/Cross-Cut Test

**Figure A4:**
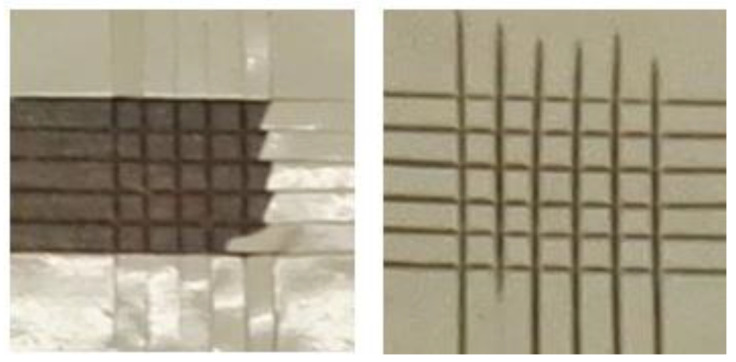
Exemplary results of the cross-cut test according to DIN EN ISO 2409 of PP60 before (left) and after (right) plasma treatment with synthetic air. The reference is assigned to class 0, the plasma-treated one, due to the still completely intact grid, to class 5.

The cross-cut test according to DIN EN ISO 2409 allows the classification of the coating’s adhesion into six different classes (0–5). While 5 means that no coating remains on the surface and the cross-cut grid is no longer visible, 0 represents the class in which the coating is still completely intact and the cut-in grid structure remains unaltered. To visualize these effects, two comparison photographs can be found in Figure A4 in the Appendix A. The results can be found in Table 3.

Therefore, the test did not allow differentiating between the working gases, but it shows the generally good adhesion of the coating after plasma treatment.

**Figure 6:**
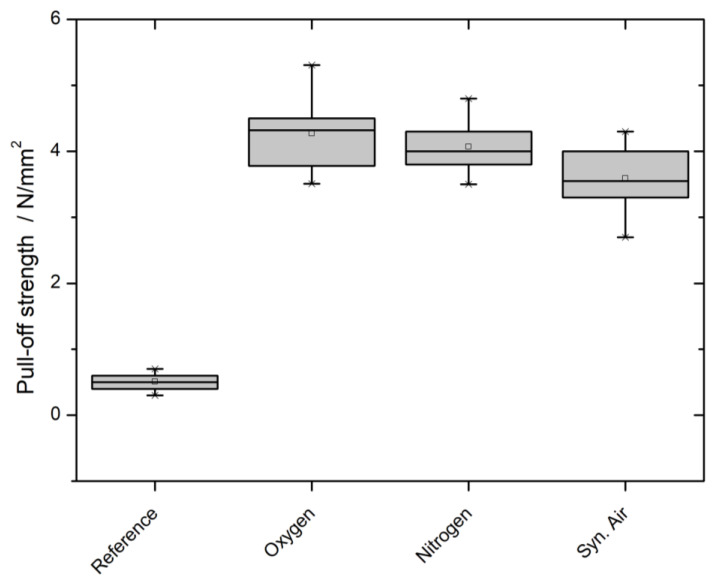
Pull-off strength of an acrylic dispersion measured according to ASTM D4541-02 (2009) and DIN EN ISO 4624 (2016) of PP60 before and after plasma treatment.

ASTM D4541-02 (2009) and DIN EN ISO 4624 (2016) both describe a pull-off test of circular-shaped dollies in which the pull-off strength is quantitatively measured using an extraction device. Due to the quantitative character of the measurement, a differentiation between the three different working gases was possible, as visualized in Figure 6.

In this case, the working gases can be differentiated. The oxygen and nitrogen plasma treatment show difference regarding the pull-off strength. In contrast, the treatment with synthetic air plasma leads to a significantly lower pull-off strength.

## 4. Discussion

Analyzing the surface chemistry confirmed previous findings of other researchers that the WPC specimen’s surface is mainly based on the matrix material properties [33,34]. Very minor peaks, in the FTIR spectrum at 3700 cm^−1^ and for XPS at 286.6 eV of the untreated references, can be attributed to hydroxyl groups. These groups are a sign of either water molecules trapped on the surface and/or a slight oxidation of the surface [35,36].

The chemical analysis after the treatment showed the generation of oxygen-based functional groups on the surface: hydroxyl, carbonyl, and carboxyl groups. The FTIR spectra (Figure 1) reveal a new peak at 1750 cm^−1^, which is very specific to double bound oxygen croups on carbon. One has to bear in mind that only new peaks provide reliable FTIR data. A quantification of FTIR data is discussed controversially and would rely on advanced statistical methods. This new peak is supported by the new peaks in the XPS spectrum after treatment (Figure 3). Notably, the surface chemistry after treatment does not differ significantly for any of the treatment variants; therefore, it is independent from the working gas used. Even though one of the working gases only consisted of argon and nitrogen, no nitrogen-based functional groups (e.g., amides, amines, imines) were detected. The formation of intermediate nitrogen groups that get substituted by oxygen on alkene substrates are reported by, e.g., Gerenser et al. and Wang et al. [37,38]. The experimental setup used in this study offers potential for this substitution throughout the experiment: the plasma chamber is operated at ambient pressure with ambient air, transport of the specimens after the treatment, and lastly the FTIR analysis itself is conducted in ambient conditions (XPS is done under ultra-high vacuum conditions). The substitution with oxygen in ambient air cannot be avoided due to technical reasons in the involved departments. However, all analysis is performed immediately after the plasma treatment to prevent a reported recombination of plasma-treated surfaces, even though the reported time scale is much higher and would allow storing [39].

The contact angle measurements of water on the WPC specimens showed the expected results: the high initial contact angle was significantly lowered (Figure 4). However, the treatments with Ar/N_2_ and Ar/O_2_ as the working gas reduced the water contact angle significantly more than the treatment with synthetic air. As described in Section 2.3.3., the contact angle can be used to calculate the surface free energy (SFE) of a material. In the equation by the approach of OWRK, the water contact angle is used to calculate the polar part of the SFE. Considering the surface chemistry analysis results, the findings are in agreement with the reduction of the water contact angle. The newly generated functional oxygen groups add a permanent dipole to the specimen’s surface. This dipole is used to form hydrogen bonds with the water molecules, increasing the wettability and therefore lowering the contact angle.

The second liquid diiodomethane is used to determine the disperse part of the SFE. This part describes only temporary, weak, and induced dipoles, as summarized in the London dispersion force. The observed slight decrease in the diiodomethane contact angle would correspond to a higher contribution to the SFE. However, due to the nature of the London disperse force, for an increase, the polymer’s chain would have to grow [40]. This is not supported by the chemical analysis data nor a logical conclusion. If any, a chain length reduction due to the plasma treatment would be expected. Looking at the results of both liquids after the plasma treatment, it is evident that the results for Ar/N_2_ and Ar/O_2_ do not differ significantly. However, in both cases, the contact angle after treatment with synthetic air is significantly higher. The effects that are analyzed by water and diiodomethane contact angle are completely contrary to each other, but the observed results on both are very comparable. A measurement artifact could be an explanation for the observed results and trends: the surface roughness is reported to have an impact on the contact angle and would affect both liquids [41]. Given the similarity of the chemical analysis results, a significant difference in the contact angles is unexpected and might be attributed to the influence of the surface morphology.

The LSM results support the hypothesis of the surface roughness as an explanation for the obtained contact angle data. In general, all three plasma treatment variants increased the surface roughness (Figure 5). The Sdr value, calculated according to DIN EN ISO 25178, describes the amount of area that is added to the base area due to the surface’s roughness. Since the specimens were measured before and after the treatment, a meaningful percentual area growth based on the corresponding references is possible (Table 2). Obviously, Ar/N_2_ and Ar/O_2_ have a comparable percentual increase of the Sdr value, while the increase for the synthetic air treatment is noticeably lower. The sputter characteristics of Ar plasma is known and well reported on different material substrates. However, comparative studies reveal an unclear picture of the question regarding which working gas is more efficiently roughening the surface. The outcome is eminently dependent on the plasma parameters, especially the treatment time [42,43,44,45,46,47,48]. A higher obtained surface roughness after the plasma treatment with the parameters described in Section 2.2 and Ar-containing gases is plausible, especially since the net treatment time is relatively short with only 30 s. This provides a conceivable explanation for the observed measurement artifacts of the contact angle measurements.

The more applied approach to investigate the adhesion of a conventional commercial acrylic dispersion coating revealed a very good adhesion. The cross-cut test (DIN EN ISO 2409) assigned the untreated reference into class 5. The coating did not adhere to the surface at all. After treatment, all three plasma working gas variants lead to the highest class 0. The newly generated oxygen functional groups offer permanent dipoles to which the coating can non-covalently hydrogen bond. The quantifiable pull-off test with 20 mm dollies (ASTM D4541-02, DIN EN ISO 4624) revealed a high pull-off strength around and exceeding 4 N/mm^2^. The previously described phenomena that Ar-containing treatments perform significantly different as synthetic air continues. These results mirror the ones obtained by contact angle and LSM measurements. They are in good agreement and consistent with the postulated hypothesis that a higher surface roughening is present and influencing other analysis methods. Due to the higher surface roughness, the coating has a higher area and therefore more possible partners with which to form a hydrogen bond.

## 5. Conclusions

A very good adhesion of common acrylic dispersion was achieved independently from the working gas that was used for the plasma treatment of PP60. The achieved 4 MPa is an exceptionally high result, promising that glue or other coatings (e.g., alkyd based) will perform well, too.

Since synthetic air, which was used to simulate well-defined ambient air, performed on par with the far more expensive Ar/N_2_ and Ar/O_2_ from a practical point of view, it appears quite clear that an industrial use case will mostly focus on compressed air. The economic benefits of availability, costs, and ease of application favor compressed air as the most viable option.

From a scientific point of view, a differentiation of the plasma treatments with different working gases is observable. While the surface chemistry of PP60 does not show significant differences between the three treatments, the surface morphology does.

Due to the assumed increased sputtering of the argon-containing working gases, the surfaces of those plasma treatments containing Ar/N_2_ and Ar/O_2_ showed a significantly increased percentual growth of surface area compared to the synthetic air treatment. This could also be shown secondarily in the increase of pull-off strength, and it explains the measurement artifacts of the contact angles.

Since an improvement due to a different working gas could be shown, future experiments can start to benchmark different working gas compositions containing argon and treatment times to improve the effect even further. With additional improvements, a use case might be found in which the higher performance compensates for the higher cost.

## Figures and Tables

**Table 1:** Elemental composition of WPC specimens surfaces (X-ray photoelectron spectroscopy (XPS) measurement) with 60% wood content in PP before and after plasma treatment with different working gases.

Working Gas	Carbon/at%	Oxygen/at%	Nitrogen/at%
Reference	98.2	1.7	0.1
Nitrogen (Ar/N_2_)	80.9	18.3	0.8
Oxygen (Ar/O_2_)	77.7	21.8	0.5
Syn. air	79.5	19.7	0.8

**Table 2:** Percentual developed interfacial area ratio (Sdr) increase of PP60 before and after plasma treatment with different working gases.

Working Gas	Sdr Increase/%
Syn. air	179.6
Oxygen (Ar/O_2_)	292.2
Nitrogen (Ar/N_2_)	304.7

**Table 3:** Cross-cut classification according to DIN EN ISO 2409 of PP60 before and after plasma treatment with different working gases.

Working Gas	Cross-Cut Classification
(Reference)	5
Syn. air	0
Oxygen (Ar/O_2_)	0
Nitrogen (Ar/N_2_)	0

## Appendix A

**Table A1:** XPS data of the C1s carbon peak from fitted Voigt profiles of PP60 before and after plasma treatment with different working gases.

Working Gas	C–C/at%	C–O/at%	C=O/at%	O–C=O/at%
Reference	96.5	3.5		
Nitrogen (Ar/N_2_)	78.8	8.6	5.0	7.6
Oxygen (Ar/O_2_)	79.9	8.1	5.0	7.0
Syn. air	80.3	8.0	4.8	6.9

## References

[1] Sobczak L., Bruggemann O., Putz R.F. (2012). Polyolefin composites with natural fibers and wood-modification of the fiber/filler-matrix interaction. J. Appl. Polym. Sci..

[2] Sobczak L., Lang R.W., Haider A. (2012). Polypropylene composites with natural fibers and wood—General mechanical property profiles. Compos. Sci. Technol..

[3] Joshi S.V., Drzal L.T., Mohanty A.K., Arora S. (2004). Are natural fiber composites environmentally superior to glass fiber reinforced composites?. Compos. Part A Appl. Sci. Manuf..

[4] Feifel S., Stübs O., Seibert K., Hartl J. (2015). Comparing wood–polymer composites with solid wood: The case of sustainability of terrace flooring. Eur. J. Wood Prod..

[5] Bütün F.Y., Sauerbier P., Militz H., Mai C. (2019). The effect of fibreboard (MDF) disintegration technique on wood polymer composites (WPC) produced with recovered wood particles. Compos. A Appl. Sci. Manuf..

[6] Krause K.C., Sauerbier P., Koddenberg T., Krause A. (2018). Utilization of Recycled Material Sources for Wood-Polypropylene Composites: Effect on Internal Composite Structure, Particle Characteristics and Physico-Mechanical Properties. Fibers.

[7] Carus M., Partanen A. (2020). Bioverbundwerkstoffe-Naturfaserverstärkte Kunststoffe (NFK) und Holz-Polymer-Werkstoffe (WPC). Fachagentur Nachwachsende Rohstoffe.

[8] Partanen A., Carus M. (2016). Wood and natural fiber composites current trend in consumer goods and automotive parts. Reinf. Plast..

[9] Chodák I., Novak I., Karger-Kocsis J. (1999). Surface modification of polypropylene by additives. Polypropylene.

[10] Brewis D.M., Briggs D. (1981). Adhesion to polyethylene and polypropylene. Polymer.

[11] Zielonka A. (2009). Jahrbuch Oberflächentechnik Band 58, 2002.

[12] Pethrick R.A., Strobel M., Lyons C.S., Mittal K.L. (1994). Plasma surface modification of polymers: Relevance to adhesion. Polymer International.

[13] Jamali A., Evans P.D. (2011). Etching of wood surfaces by glow discharge plasma. Wood Sci. Technol..

[14] Wolkenhauer A., Avramidis G., Cai Y., Militz H., Viöl W. (2007). Investigation of wood and timber surface modification by dielectric barrier discharge at atmospheric pressure. Plasma Process. Polym..

[15] Wascher R., Leike N., Avramidis G., Wolkenhauer A., Militz H., Viöl W. (2015). Improved DMDHEU uptake of beech veneers after plasma treatment at atmospheric pressure. Eur. Wood Wood Prod..

[16] Köhler R., Sauerbier P., Militz H., Viöl W. (2017). Atmospheric Pressure Plasma Coating of Wood and MDF with Polyester Powder. Coatings.

[17] Hünnekens B., Avramidis G., Ohms G., Krause A., Viöl W., Militz H. (2018). Impact of plasma treatment under atmospheric pressure on surface chemistry and surface morphology of extruded and injection-molded wood-polymer composites (WPC). Appl. Surf. Sci..

[18] Oporto G.S., Gardner D.J., Bernhardt G., Neivandt D.J. (2007). Characterizing the mechanism of improved adhesion of modified wood plastic composite (WPC) surfaces. J. Adhes. Sci. Technol..

[19] Oporto G.S., Gardner D.J., Bernhardt G., Neivandt D.J. (2009). Forced Air Plasma Treatment (FAPT) of Hybrid Wood Plastic Composite (WPC)-Fiber Reinforced Plastic (FRP) Surfaces. Compos. Interfaces.

[20] Sauerbier P., Anderson J., Gardner D.J. (2018). Surface Preparation and Treatment for Large-Scale 3D-Printed Composite Tooling Coating Adhesion. Coatings.

[21] Ten Bosch L., Pfohl K., Avramidis G., Wieneke S., Viöl W., Karlovsky P. (2017). Plasma-Based Degradation of Mycotoxins Produced by Fusarium, Aspergillus and Alternaria Species. Toxins.

[22] Krüss GmbH: Measuring with Method—But with Which One?. https://www.kruss-scientific.com/fileadmin/user_upload/website/literature/kruss-tn314-en.pdf.

[23] Kaelble D.H. (1970). Dispersion-Polar Surface Tension Properties of Organic Solids. J. Adhes..

[24] Owens D.K., Wendt R.C. (1969). Estimation of the surface free energy of polymers. J. Appl. Polym. Sci..

[25] Owens, Wendt, Rabel and Kaelble (OWRK) Method. https://www.kruss-scientific.com/services/education-theory/glossary/owens-wendt-rabel-and-kaelble-owrk-method/.

[26] European Committee for Standardization (2012). DIN EN ISO 25178-2:2012-09 Geometrical Product Specifications (GPS)-Surface Texture: Areal-Part 2: Terms, Definitions and Surface Texture Parameters (ISO 25178-2:2012).

[27] ASTM International (2002). ASTM D4541-02 Standard Test Method for Pull-Off Strength of Coatings Using Portable Adhesion Testers.

[28] European Committee for Standardization (2016). DIN EN ISO 4624:2016-08 Paints and Varnishes-Pull-Off Test for Adhesion (ISO 4624:2016).

[29] European Committee for Standardization (2016). DIN EN ISO 2409:2013-06 Paints and Varnishes-Cross-Cut Test (ISO 2409:2013).

[30] Beamson G., Briggs D. (1992). High Resolution XPS of Organic Polymers: The Scienta ESCA300 Database.

[31] Morent R., De Geyter N., Leys C., Gengembre L., Payen E. (2008). Comparison between XPS- and FTIR-analysis of plasma-treated polypropylene film surfaces. Surf. Interf. Anal..

[32] Jaleh B., Parvin P., Wanichapichart P., Saffar A.P., Reyhani A. (2010). Induced super hydrophilicity due to surface modification of polypropylene membrane treated by O_2_ plasma. Appl. Surf. Sci..

[33] Akhtarkhavari A., Kortschot M.T., Spelt J.K. (2004). Adhesion and durability of latex paint on wood fiber reinforced polyethylene. Prog. Org. Coat..

[34] Hünnekens B., Peters F., Avramidis G., Krause A., Militz H., Viöl W. (2016). Plasma treatment of wood–polymer composites: A comparison of three different discharge types and their effect on surface properties. J. Appl. Polym. Sci..

[35] Hadjiivanov K. (2014). Identification and Characterization of Surface Hydroxyl Groups by Infrared Spectroscopy. Advances in Catalysis.

[36] Fabiyi J.S., McDonald A.G., Wolcott M.P., Griffiths P.R. (2008). Wood plastic composites weathering: Visual appearance and chemical changes. Polym. Degrad. Stab..

[37] Gerenser L.J. (1987). X-Ray photoemission study of plasma modified polyethylene surfaces. J. Adhes. Sci. Technol..

[38] Wang K., Wang W., Yang D., Huo Y., Wang D. (2010). Surface modification of polypropylene non-woven fabric using atmospheric nitrogen dielectric barrier discharge plasma. Appl. Surf. Sci..

[39] Hünnekens B., Krause A., Militz H., Viöl W. (2017). Hydrophobic recovery of atmospheric pressure plasma treated surfaces of Wood-Polymer Composites (WPC). Eur. J. Wood Wood Prod..

[40] Fowkes F.M. (1980). Surface effects of anisotropic London dispersion forces in n-alkanes. J. Phys. Chem..

[41] Busscher H.J., Van A.W.J., Bobr P.D., Jono H.P.D., Arends J. (1984). The effect of surface roughenlng of polymers on measured contact angles of liquids. Colloids Surfaces.

[42] Satake M., Iwase T., Kurihara M., Negishi N., Tada Y., Yoshida H. (2013). Effect of oxygen addition to an argon plasma on etching selectivity of poly(methyl methacrylate) to polystyrene. J. Micro/Nanolith. MEMS MOEMS.

[43] Slepička P., Vasina A., Kolská Z., Luxbacher T., Malinský P., Macková A., Švorčík V. (2010). Argon plasma irradiation of polypropylene. Nuclear Instrum. Methods Phys. Res. Sect. B Beam Interact. Mater. At..

[44] Lee S.D., Sarmadi M., Denes F., Shohet J.L. (1997). Surface modification of polypropylene under argon and oxygen-RF-plasma conditions. Plasma Pol..

[45] Terpilowski K., Rymuszka D., Holysz L., Chibowski E. Changes in wettability of polycarbonate and polypropylene pretreated with oxygen and argon plasma. Proceedings of the 8th International Conference MMT-20142.

[46] Seo E.-D. (2004). Atomic force microscopy and specular reflectance infrared spectroscopic studies of the surface structure of polypropylene treated with argon and oxygen plasmas. Macromol. Res..

[47] Spyrides S.M.M., Alencastro F.S., Guimaraes E.F., Bastian F.L., Simao R.A. (2019). Mechanism of oxygen and argon low pressure plasma etching on polyethylene (UHMWPE). Surf. Coat. Technol..

[48] James J., Joseph B., Shaji A., Nancy P., Kalarikkal N., Thomas S., Grohens Y., Vignaud G. (2019). Microscopic Analysis of Plasma-Activated Polymeric Materials. Non-Thermal Plasma Technology for Polymeric Materials.

